# History of the Ophthalmoscope

**DOI:** 10.7759/cureus.66387

**Published:** 2024-08-07

**Authors:** Anish Amirneni, Garv Bhasin, Latha Ganti

**Affiliations:** 1 Biomedical Sciences, University of Central Florida, Orlando, USA; 2 Biology, Brown University, Providence, USA; 3 Emergency Medicine & Neurology, University of Central Florida, Orlando, USA; 4 Research, Orlando College of Osteopathic Medicine, Winter Garden, USA; 5 Medical Science, The Warren Alpert Medical School of Brown University, Providence, USA

**Keywords:** eye exam, general ophthalmology, glaucoma, ophthalmoscope, retina

## Abstract

This article will describe the history of an important tool: the ophthalmoscope. The evolution of the ophthalmoscope will be analyzed, beginning with its first historical reference and detailing every iteration of the instrument leading up to the version we know today. This article will elaborate on the significance of the ophthalmoscope as a vital instrument for performing eye fundus examinations and explain why it is a staple accouterment in the field of ophthalmology.

## Introduction and background

**Figure 1:**
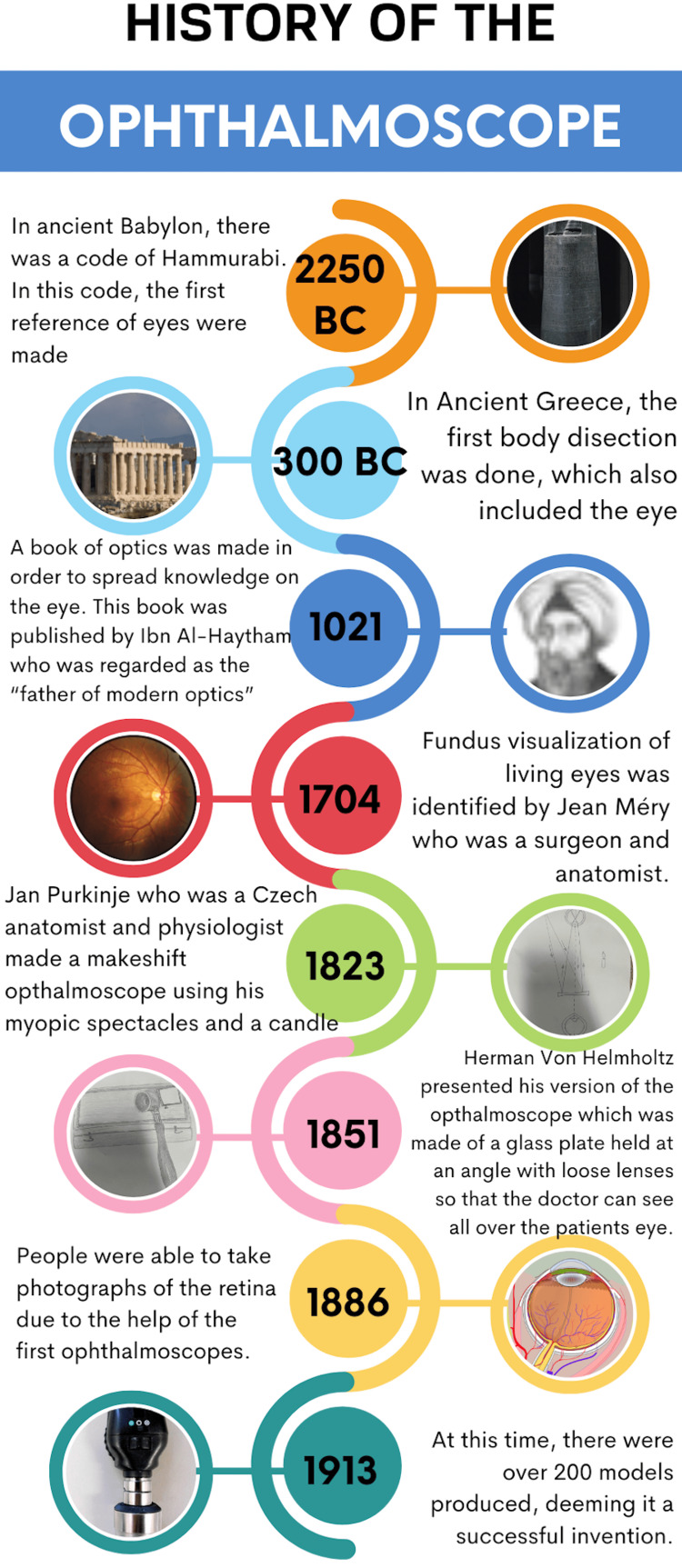
History of the ophthalmoscope (infographic designed by Anish Amirneni on Canva.com) All images in the infographic are from Creative Commons sources [3].

The ophthalmoscope is an instrument that is used to inspect the retina and posterior segment of a patient’s eye. Physicians always have their ophthalmoscope in arm's reach due to its importance in everyday checkups. Before the time of the ophthalmoscope in the early 1800s, it was impossible for doctors to analyze the posterior segment of a patient's eye while the patient was still conscious. The ophthalmoscope would eventually provide a noninvasive, quick, and easy mechanism for the examination of the posterior segment of the eye. This instrument is necessary to find and evaluate signs and symptoms of potential vision-threatening pathologies. Some of the complications that can be detected by the ophthalmoscope are glaucoma (a condition in which the optic nerve is damaged potentially causing blindness), retinal detachment (where the retina gets detached from its supportive issue), optic nerve hypoplasia (a condition where the optic nerve does not develop properly), and melanoma of the eye (a type of cancer), among others [1]. Currently, there are various versions of the ophthalmoscope, such as the direct, indirect, and scanning laser ophthalmoscopes [2]. All of these different versions integrated new technological advancements that resulted in the modernization of optic technology. This review seeks to catalog the evolution of the ophthalmoscope and discuss its impact on medicine (Figure 1).

## Review

References to studying the eye started as early as the fourth century B.C. (before the common era) approximately 2,400 years ago. In ancient Greece, circa 300 BC, the first full body dissection was held, which included the dissection of the eye. This eye dissection signified the inception of society’s increasing recognition of the eye’s pivotal role in the human anatomy and optics. Furthermore, in the year 1021, Ibn-Al Haytham, who is regarded as “the father of optics,” published a book named “Kitab al Manazir” which translated to “Book of Optics”. In it, he describes the theory of vision based on light emanating from objects [4]. Haytham published this book in order to spread knowledge about ophthalmology [5]. These events underscore the burgeoning importance of studying the eye and its anatomy in ancient society.

Jan Purkinje, a Czech anatomist and physicist who is famous for discovering the Purkinje Fibers in the heart, was a major contributor to the field of ophthalmology. Purkinje exhibited a technique to examine the fundus of the eye long before Hermann Von Helmholtz, who was eventually deemed the inventor of the ophthalmoscope [6]. In the year 1823, Purkinje manufactured a makeshift ophthalmoscope using only myopic spectacles and a candle [7]. Unfortunately, Purkinje's work was disregarded by his peers, who gave credit for the creation of the first ophthalmoscope to Helmholtz [6]. 

The first version of the ophthalmoscope to be invented was the direct ophthalmoscope. This was a rudimentary version of the ophthalmoscope; however, this preliminary version of the ophthalmoscope was what led to more inventions and was the building block of the examination of the eye. Hermann Von Helmholtz, a prolific physicist and physician, initially spent most of his life as a general scientist. Helmholtz’s father encouraged him to study medicine and specialize to become a physician. Helmholtz eventually became a physicist for the military, but after several years, he pivoted to become a professor in ocular physiology in the year 1848. Finally, in 1850, Helmholtz created the first documented version of the ophthalmoscope [7]. His initial experiment consisted of looking through a glass plate at a specific angle, allowing the light to be reflected into his own eye [8]. Afterward, he used the ophthalmoscope to create images of his eye using the rays of the reflecting light. In Helmholtz’s first use of the ophthalmoscope, he made discoveries such as how the pupil normally looks black, but under certain light conditions, the pupil seemed to turn red and brighter than normal. Helmholtz was inspired by his findings and began to create optical images that monitored blood vessel growth in tumors and he also measured properties of the eye tissue. Through these innovations, Helmholtz influenced many other researchers in countries around Europe, such as Germany, Austria, and the Netherlands, and laid the groundwork for future breakthroughs in the fascinating field of ophthalmology. Soon, the relevance of the ophthalmoscope began to rise as it was increasingly popularized by scientists and global leaders. At the first International Congress of Ophthalmology in Brussels held in 1857, the representatives of the Congress recognized the importance of ophthalmoscopes and their role in the future of medicine [9]. 

Another version of the ophthalmoscope made by Felix Giraud-Teulon was constructed in 1861. The direct ophthalmoscope was relatively limited in its field of view, and as such, Giraud-Teulon wished to enhance the direct ophthalmoscope by modifying the instrument to expand the field of view of the eye. Giraud-Teulon’s version of the ophthalmoscope is considered better than its predecessor because it includes a larger field view of the eye, aiding the physician in performing a more thorough analysis of the eye. In addition to the augmented field of view, the newer version of the ophthalmoscope attempts to provide more depth of focus and a larger image of the posterior segment of the eye itself when scanning and examining it. To accomplish this, Giraud-Teulon integrated a magnifying glass into the ophthalmoscope instead of a mirror. The magnifying glass was connected to a flexible arm that was attached to the head of the instrument. The combination of the increased maneuverability provided by the arm and the clarity provided by the magnifying glass gave the ophthalmoscope more perspective and angles for examining the eye. One of the main reasons for this improvement was to enable doctors or physicians to examine the fundus, and the posterior pole of the eye, which was difficult to accomplish given the limited field of view of the direct ophthalmoscope [10]. 

The next big developments were concerned with improving lighting conditions as it would allow for a more thorough eye examination and better detection of malignancies. In 1869, the gravity-fed oil lamp (a lamp that harnesses the force of gravity to bring the oil to the wick of the lamp), and the gas-burning lamp (consisting of incandescent lights) were introduced to enhance the use of light during the eye examination [7]. Furthermore, in 1885, William Dennet, a New York ophthalmologist, implemented an electric light bulb into his ophthalmologic operations. Although many people thought that this was a good idea, it did not work due to the small life of the light bulb and its unreliability in daily use [6]. Again, in 1886, Thomas Reid, an ophthalmologist from Scotland, modified the column of the instrument and replaced the mirror with a prism to project the light into the eye. Similar to Dennet, this model was not successful and it did not go into production. A few months afterward in 1886, Henry Juler, an author from England, revamped the ophthalmoscope by successfully adding a light bulb inside the instrument. This version of the ophthalmoscope was feasible for production as the life of the light bulb at this time was longer than the electric light bulb that Dennet had implemented. 

Another version of the ophthalmoscope was introduced by Robert Webb in 1980 and was called the scanning laser ophthalmoscope. This ophthalmoscope is different from the other versions because it uses a collimated (parallel) beam of laser light instead of a light bulb [11]. The laser ophthalmoscope is not as common as the direct or indirect ophthalmoscope versions, but it is still significant as it utilizes multi-wavelength illumination technology. This technology emits light at different wavelengths that are absorbed by different parts of the eye which allows for targeted examination and treatment of specific tissues. The scanning laser ophthalmoscope provides faster, higher-contrast images of the eye compared to the direct and indirect ophthalmoscopes [12]. Despite these advantages, the laser ophthalmoscope was not as widely used as its counterparts due to its excessive price and bulky equipment.

Currently, the direct ophthalmoscope is the most popular and iconic version of the ophthalmoscope. Although more peripheral retina parts of the eye can be seen with a larger field of view using the indirect ophthalmoscope, doctors prefer the use of the direct version because of its 15 times magnified view. The greater magnification provides a better view of venous pulsations and vascular and optic nerve changes in the eye. The direct ophthalmoscope is also more accessible as it is possible to be carried in the physician’s coat pocket. Comparatively, the indirect or laser ophthalmoscopes are larger instruments that are more tedious to set up, and as such, their usage may not be best suited for the fast-paced nature of the physical checkup [13].

## Conclusions

The ophthalmoscope has had a profound impact on medicine. It has allowed scientists to study the anatomy of the inside of the eye, a part of the body that was previously impossible to study in the living. Over 200 years, the ophthalmoscope evolved from using unsophisticated objects, such as candles, mirrors, and prisms, to using more advanced technology, such as lasers, magnifying glasses, and lightbulbs. The ophthalmoscope has literally and figuratively opened our eyes by enabling us to understand a physiological system that is so fundamental to our existence.
